# Biomechanical assessment of different fixation methods in mandibular high sagittal oblique osteotomy using a three-dimensional finite element analysis model

**DOI:** 10.1038/s41598-021-88332-2

**Published:** 2021-04-22

**Authors:** Charles Savoldelli, Elodie Ehrmann, Yannick Tillier

**Affiliations:** 1 Department of Oral and Maxillofacial Surgery, Head and Neck Institute, University Hospital of Nice, 30 Avenue Valombrose, 06100 Nice, France; 2 Department of Computational Mechanics Physics CEMEF, MINES ParisTech, PSL Research University, Centre de Mise en Forme Des Matériaux (CEMEF), French National Centre for Scientific Research, Sophia Antipolis, France; 3 Department of Orthodontics, Oral Rehabilitation and Facial Pain, Dentistry Unit, University Hospital of Nice, Nice, France

**Keywords:** Health care, Medical research, Engineering, Mathematics and computing

## Abstract

With modern-day technical advances, high sagittal oblique osteotomy (HSOO) of the mandible was recently described as an alternative to bilateral sagittal split osteotomy for the correction of mandibular skeletal deformities. However, neither in vitro nor numerical biomechanical assessments have evaluated the performance of fixation methods in HSOO. The aim of this study was to compare the biomechanical characteristics and stress distribution in bone and osteosynthesis fixations when using different designs and placing configurations, in order to determine a favourable plating method. We established two finite element models of HSOO with advancement (T1) and set-back (T2) movements of the mandible. Six different configurations of fixation of the ramus, progressively loaded by a constant force, were assessed for each model. The von Mises stress distribution in fixations and in bone, and bony segment displacement, were analysed. The lowest mechanical stresses and minimal gradient of displacement between the proximal and distal bony segments were detected in the combined one-third anterior- and posterior-positioned double mini-plate T1 and T2 models. This suggests that the appropriate method to correct mandibular deformities in HSOO surgery is with use of double mini-plates positioned in the anterior one-third and posterior one-third between the bony segments of the ramus.

## Introduction

**Figure 1:**
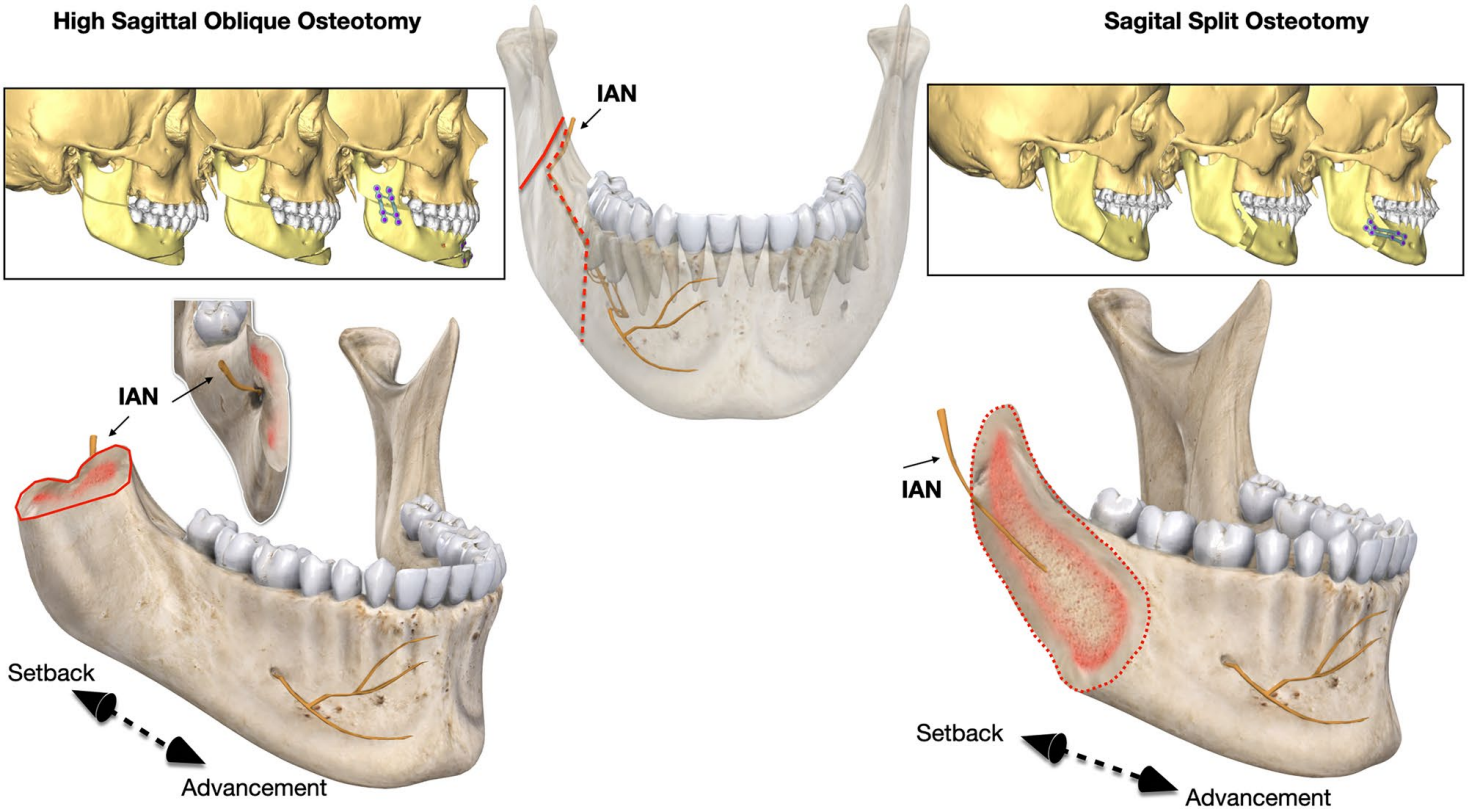
Schematic comparison of high sagittal split osteotomy (HSOO, left) and bilateral sagittal split osteotomy (BSSO, right).

Bilateral sagittal split osteotomy (BSSO) is a surgical technique commonly used to correct mandibular deformities. Combined with orthodontic treatment, this technique provides a satisfactory occlusal relationship and facial appearance. However, the main complication of this procedure remains neurosensory disturbance with sensitivity deficit of the chin and lower labial area due to permanent injury to the inferior alveolar nerve^1,2^. High sagittal oblique osteotomy (HSOO**)** of the mandible with modern-day technical advances^3^ was recently described as an alternative to BSSO to provide mandibular skeletal deformity correction and avoid surgical damage to the inferior alveolar nerve (Fig. 1, right). Previously, the classical approach to treating this type of deformity was to perform a horizontal osteotomy of the ramus. It consisted of performing a horizontal osteotomy between the sigmoid notch and the mandibular foramen^4^. This original design to the osteotomy of the ascending ramus resulted in a lack of adequate postoperative osteo-fixation, and a small contact area^4,5^. The “modern” HSOO consists of performing an osteotomy above the mandibular foramen at an average angle of 45° with respect to the vertical ramus of the mandible (Fig. 1, left). Then, the distal segment of the mandible is mobilised, placed and fixed into the planned position within an increasing segment contact after repositioning. Advancement or setback of the distal segment during HSOO depends on the mandibular deformities (retrognathism or prognathism) but both result in little contact across the osteotomy site. Ensuring a stable fixation remains a primary concern in providing bony union. Stability of HSOO is therefore determined by fixation techniques in the early postoperative phase. In general, for HSOO, surgeons have used standard^3,5^, grid-double^6^ or dedicated^5,7,8^ mini-plates to ensure an optimal fixation. The way in which they are used mainly depends on the practitioner’s experience and preferences. Many clinical and radiological studies^5,7–10^ have been conducted in HSOO patients to demonstrate the relevance of this specific technique in terms of less neurosensory disturbance^11^, but none of the studies had assessed the optimal position or design of the fixation method. Moreover, neither in vitro nor numerical biomechanical assessment was carried out to evaluate the performance of fixation methods in HSOO, despite a number of studies investigating biomechanical modifications of the described techniques of BSSO^12^. Among the biomechanical evaluation methods, the finite element analysis (FEA) model is widely used to assess the stress distribution of fixation methods occurring during different types of jaw surgery^13–16^. FEA simulates the mechanical aspect of a structure or tissue under load and the von Mises stresses can yield estimates of stress data over the entire continuum of any fixation configuration during osteosynthesis^17^. The aim of this finite element study was to compare the biomechanical characteristics and stress distribution in osteosynthesis plates when using different designs and placing configurations, in order to propose an effective plating method when undertaking HSOO surgery.

## Materials and methods

### FE model

**Figure 2:**
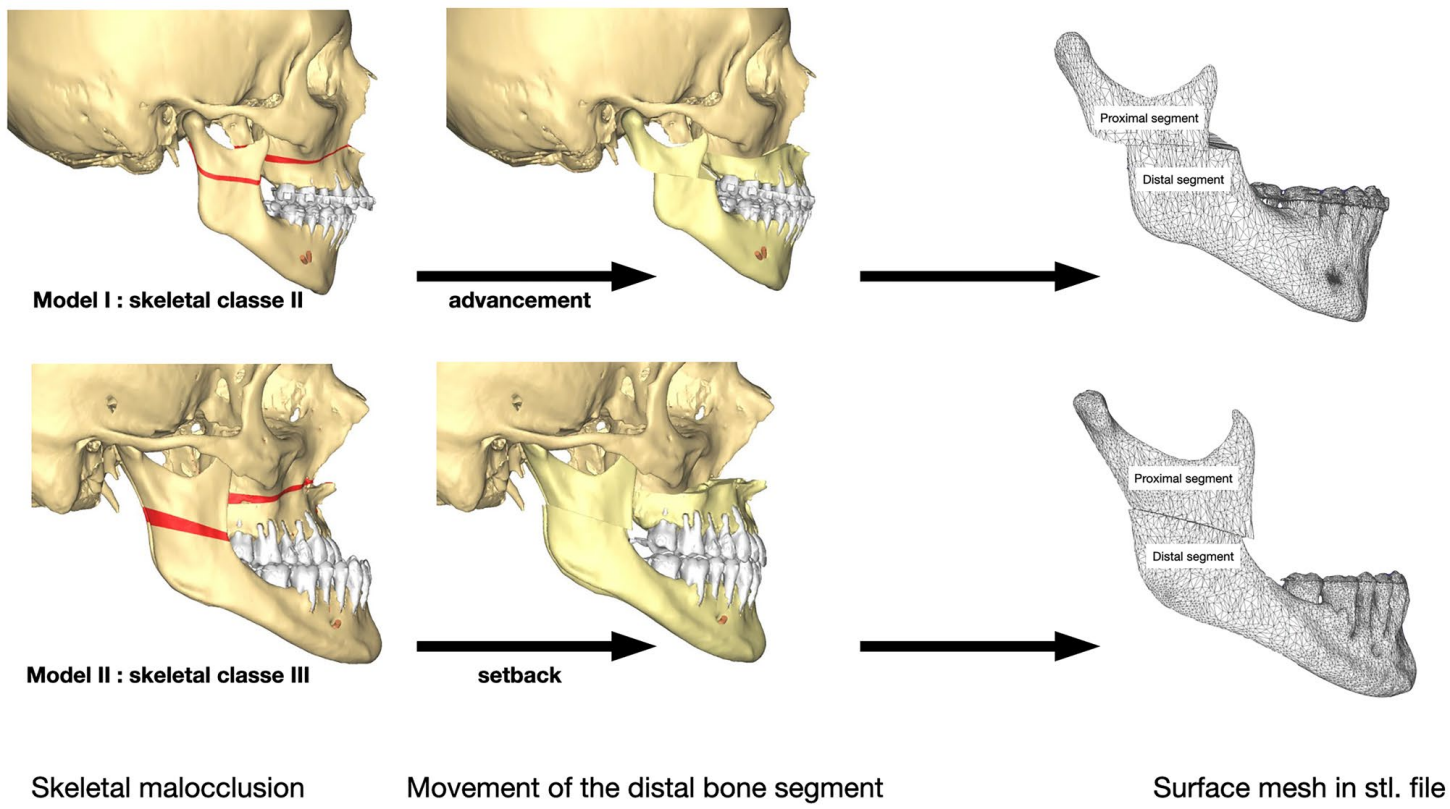
Three-dimensional T1 and T2 models obtained respectively from Class II and III skeletal malocclusion during 3D planning.

**Figure 3:**
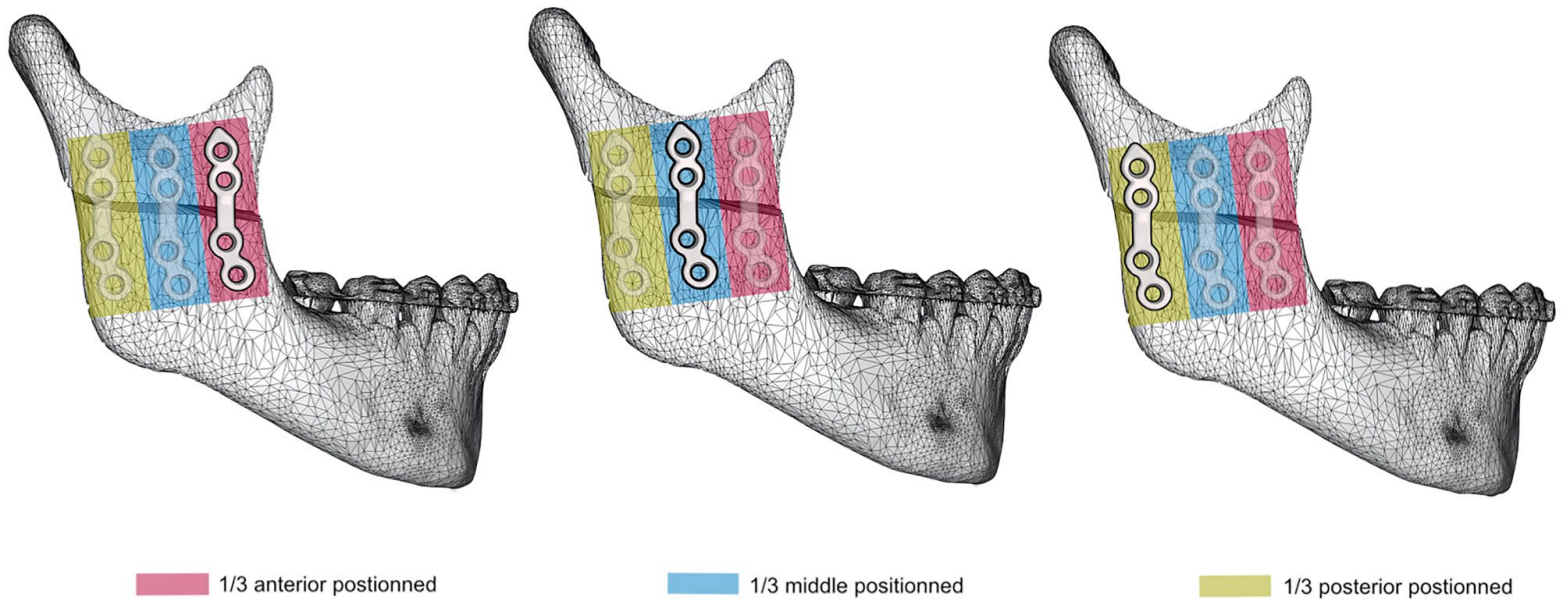
Areas available for mini-plate fixation in high oblique sagittal split osteotomy.

Two patients presenting each with a dento-skeletal class II and class III malocclusion were previously treated with HSOO. The patients underwent mandibular advancement and mandibular set-back, respectively, with numerical planning and patient specific implants for a fixation and positioning system. All methods were carried out in accordance with relevant guidelines, regulations and medical research ethics (World Medical Association Declaration of Helsinki). All experimental protocols were approved by an internal institutional review board (Nice University Head and Neck Institute), this study was declared to the “Commission Nationale Informatique et Liberté” (CNIL N° 2219288) and informed consents were obtained for this study. The computer tomography (CT) images from the surgical planning of both patients were reacquired after surgery. Three-dimensional digital modelling of half of the mandible was performed by segmenting the acquired DICOM images with a 0.65 mm thick cut using Materialise 3-matic Medical version 15.0 (Materialise, Leuven, Belgium). The segmented model was then exported as an .stl file (surface mesh) and used by ProPlan CMF (Materialise, Leuven, Belgium) to simulate the numerical HSOO, according to modern-day technical advances^3^. Two bone segments were obtained, one proximal and one distal. The distal bone segment was advanced by 5 mm in model 1 (T1) and retracted by 3 mm in model 2 (T2; Fig. 2) to comply with surgical planning. The fixation units were designed using Materialise 3-matic Medical version 15.0 (Materialise, Leuven, Belgium). For each model, six different geometries of mini-plate, with 1.2 mm thick fixation and monocortical screws, were generated so that the surgical operation could be simulated with each of them. They were designed in order to be applied in three different areas: three single plates placed vertically on the external side of ramus in the anterior one-third (A1), in the middle one-third (A2), and in the posterior one-third (A3) positions, and three double parallel bridge-plates placed vertically in the anterior and middle one-third (A4), in the anterior and posterior one-third (A5), and in the middle and posterior one-third (A6) positions of the ramus (Fig. 3). An experienced surgeon and an engineer from OBL-Materialise company (Malakoff, France) managed this initial step. Each mini-plate model was exported as a .stl file. Mini-plates and screws were assembled, smoothed, and cleaned up using the Autodesk Meshmixer free software version 3.5.374 (available at https://www.meshmixer.com) and Meshlab free software version 2016.12 (available at https://www.meshlab.net), and then exported again as surface meshes, together with those of the mandible segments (.stl files) into the FORGE NxT 3.0 finite element software (Transvalor, Biot, France) which has been already validated using different biomechanical models^18–20^.

**Figure 4:**
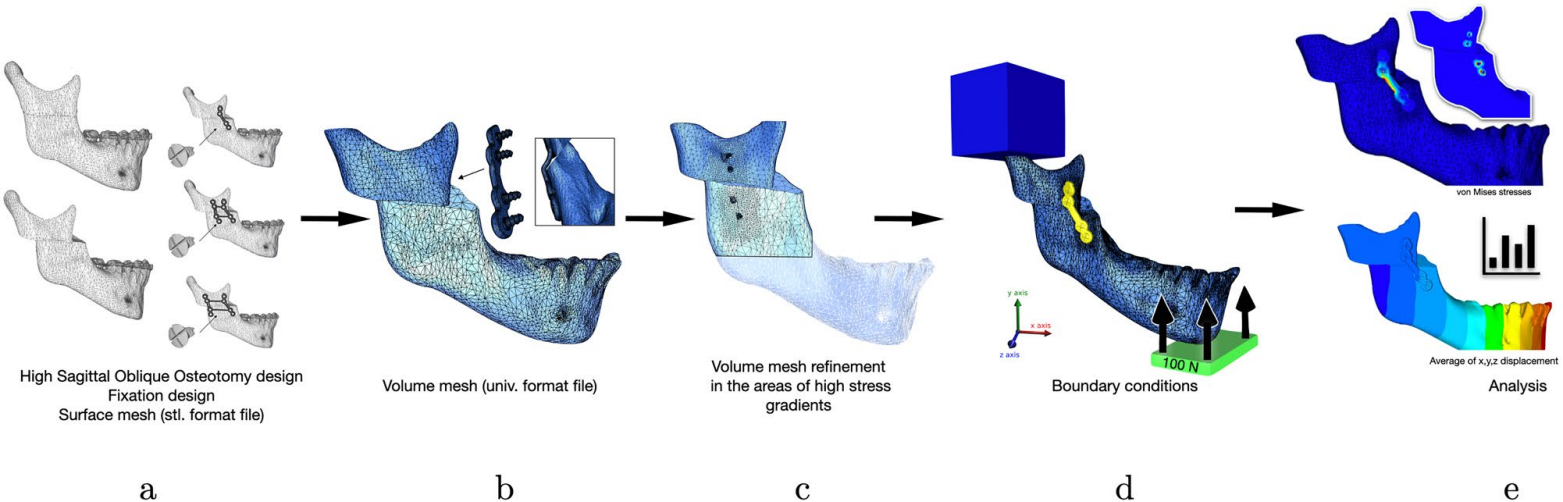
Methodology. (**a**) high sagittal oblique osteotomy and fixation design in surface mesh (stl. format file); (**b**) volume mesh generation (univ. format file); (**c**) volume mesh refinement in the areas of high stress gradients; (**d**) condyle boundary conditions and mandible loading; and (**e**) von Mises stresses and displacement analysis.

An adaptive volume meshing (I-deas Universal File format) of the different parts (mini-plates, screws and bone segments) has been created due to the volumetric mesher included in the FORGE NxT 3.0 package (GLpre). Mesh boxes were created to limit the number of elements (coarser mesh) in areas where small deformation are expected in order to reduce computing time and to refine the mesh in areas that are likely to have high strain or stress levels, such as around the screw holes in bone segments, and in the fixation devices (Fig. 4).

The final FEM model of the mandible had an average of 72,349 tetrahedral (± 1334) elements in the T1 model and tetrahedral 67,424 (± 986) in the T2 model.

The number of tetrahedral elements depended on the mesh refinement around the screw holes and on the geometry of the mini-plates (and thus on the number of screws required).

### Material properties

All materials used were assumed to be homogenous, isotropic and linear elastic. The elastic modulus (*E*) and Poisson ratio (γ) for bone and titanium alloy were assigned according to a previous study^18,21^ and are summarised in Table 1 . Coulomb’s law was used to account for friction between the different components of the model. Low friction contact was used between bone fragments to simulate a non-osseointegration and the nature of interface between bone fragments immediately after surgery ($\overline{m}$ = 0.05, μ = 0.02). The contact surface between bone segments and screw units was assumed with sticking contact, and between bone segments and plates units with sliding contact.

**Table 1:** Assigned mechanical properties of different components.

Model	Biomechanical behaviour law	Young’s modulus (*E*), MPa	Poisson’s ratio (γ)
Compact bone	Linear elasticity (Hooke’s law)	13,700	0.30
Cancellous bone	Linear elasticity (Hooke’s law)	7,930	0.35
Fixations: screw and plates (TA6V Titanium alloy)	Linear elasticity (Hooke’s law)	114,000	0.34

### Boundary conditions

Assuming that the proximal and distal segments were fixed by fixation units, an upward displacement was applied linearly and perpendicularly to the basal area of the chin, with the condylar region blocked in all directions in order to maximize the shear forces in the operated area. The displacement imposed on the jaw corresponds to a masticatory force of the order of maximum 100 N, corresponding to biting forces that patients who have undergone orthognathic surgery might encounter in the beginning of the postoperative period, and is therefore lower than in the non-operated population. The calculations were performed out on a 16 processors computer with the FORGE NxT 3.0 FE analysis program. The computed results were analysed using the Glview Inova post-processor included in the FORGE NxT 3.0 package. The evaluated parameters were the von Mises stress distribution in the fixation units, the peak of stress in bones, and the shift displacement in the gap between the proximal and distal bone segments.

## Results

**Figure 5:**
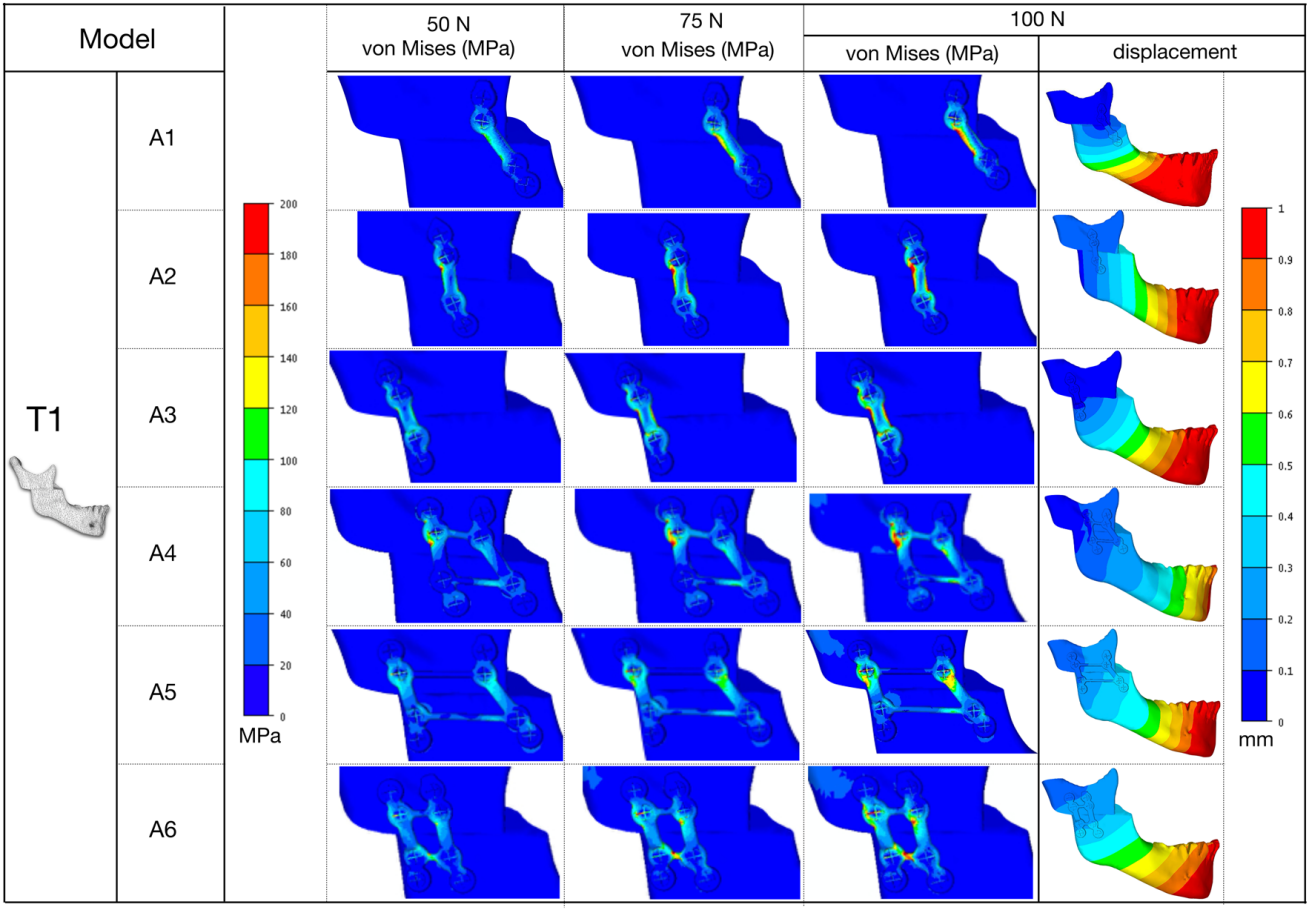
von Mises stress distribution in fixation schemes and the displacement shift between the proximal and distal bone segments in the T1 model.

**Figure 6:**
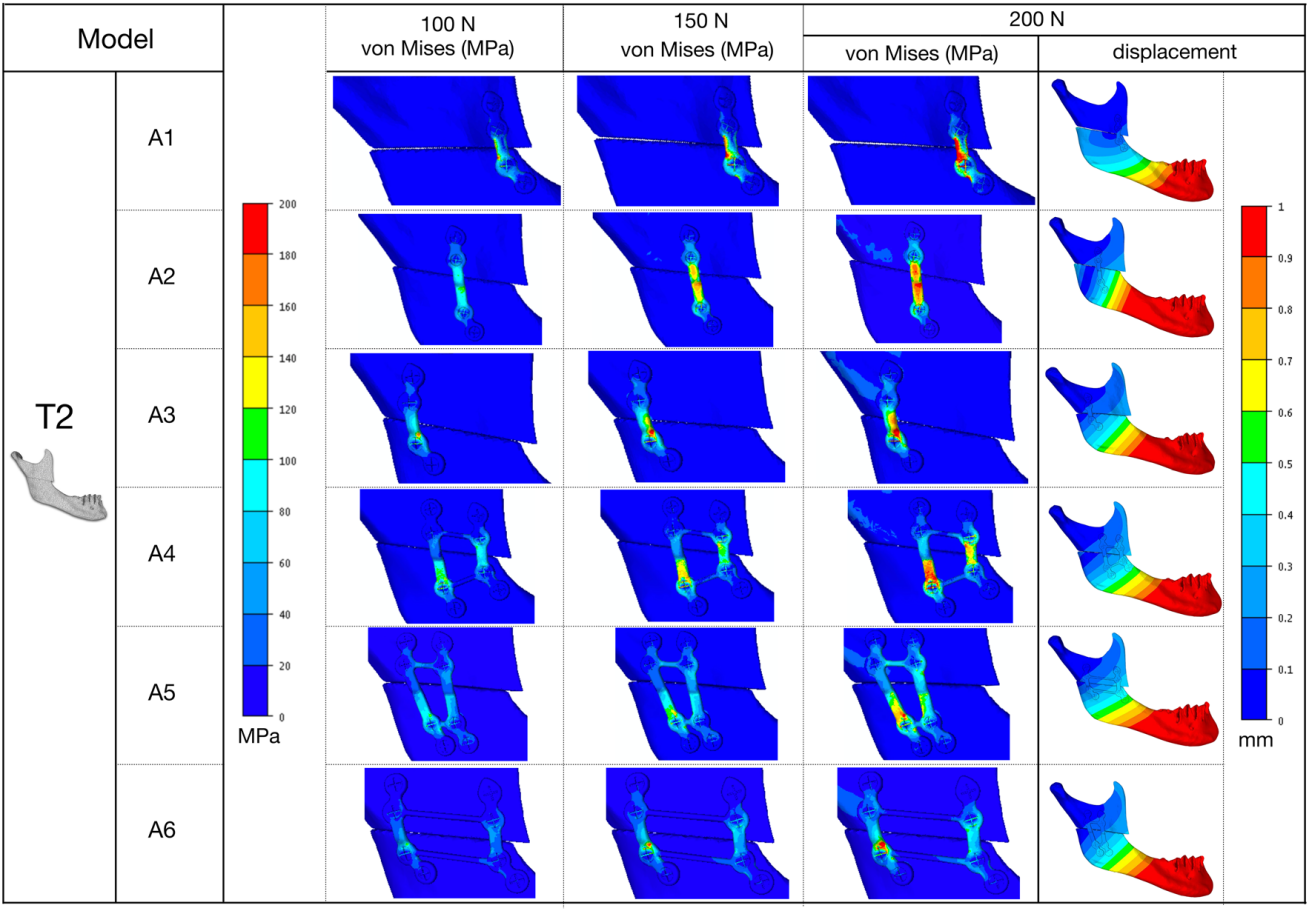
von Mises stress distribution in fixation schemes and the displacement shift between the proximal and distal bone segments in the T2 model.

**Figure 7:**
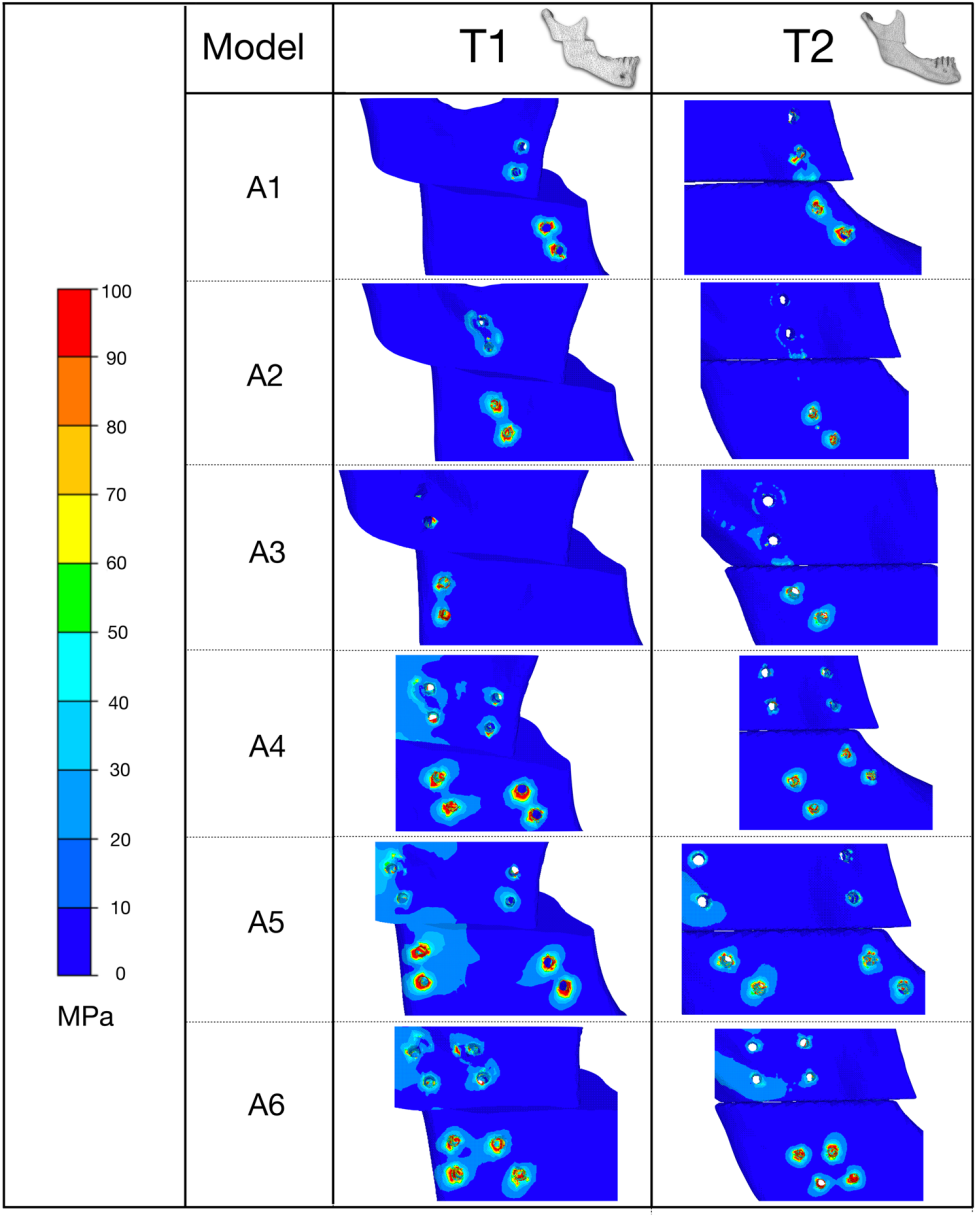
von Mises stress distribution in bone around the hole drilling under 100 N.

**Figure 8:**
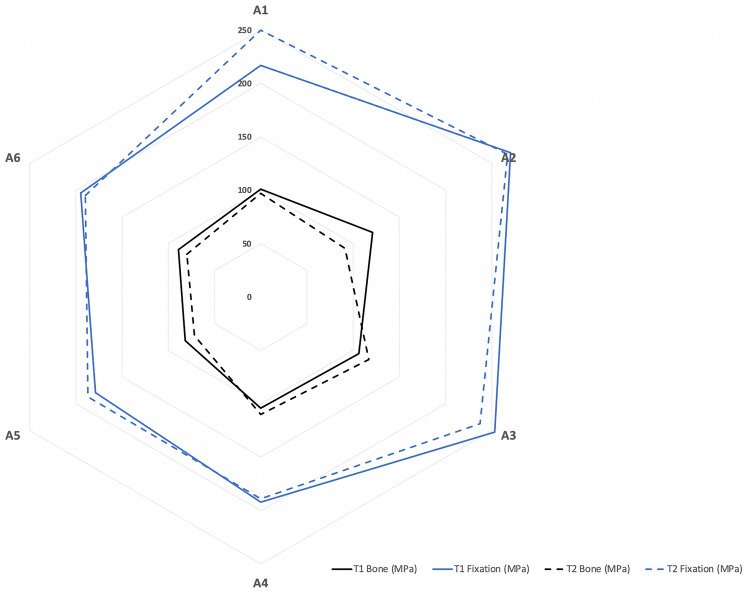
von Mises Stress peaks in bone and in fixations show the lowest stresses in the A5 configuration for both the T1 and T2 models.

Figures 5 and 6 show the von Mises stress distribution in fixation schemes at 50, 75, 100 N, and the displacement shift between the proximal and distal bone segments under 100 N in the T1 and T2 models. Figure 7 shows the von Mises stress distribution in bone around the hole drilling under 100 N, and Fig. 8 summarizes the maximal peak stress distribution under a 100 N vertical load in fixation and bone.

### Von Mises stress in fixation

The results of stress distribution in the same plating configuration for T1 and T2 showed a similar trend. The stress distribution showed a smoother location with less stress concentrated in the double-plate (A4, A5, A6) than the single-plate (A1, A2, A3) configurations. Higher von Mises stress peaks were observed in T1-A2 and T2-A1 (single-plate) models, while the T1-A5 and T2-A5 (double-plate) models showed lower stresses.

### Von Mises stress in bone

High stresses were uniformly distributed around the screw holes of both bone segments in the double-plate configurations (A4, A5, A6) but more concentrated in the distal segment in the single-plate configurations (A1, A2, A3). Higher von Mises stress peaks were observed in the T1-A2 and T2-A4 models, while the T1-A5 and T2-A5 models again showed lower stress values.

### Bone segment displacement

**Figure 9:**
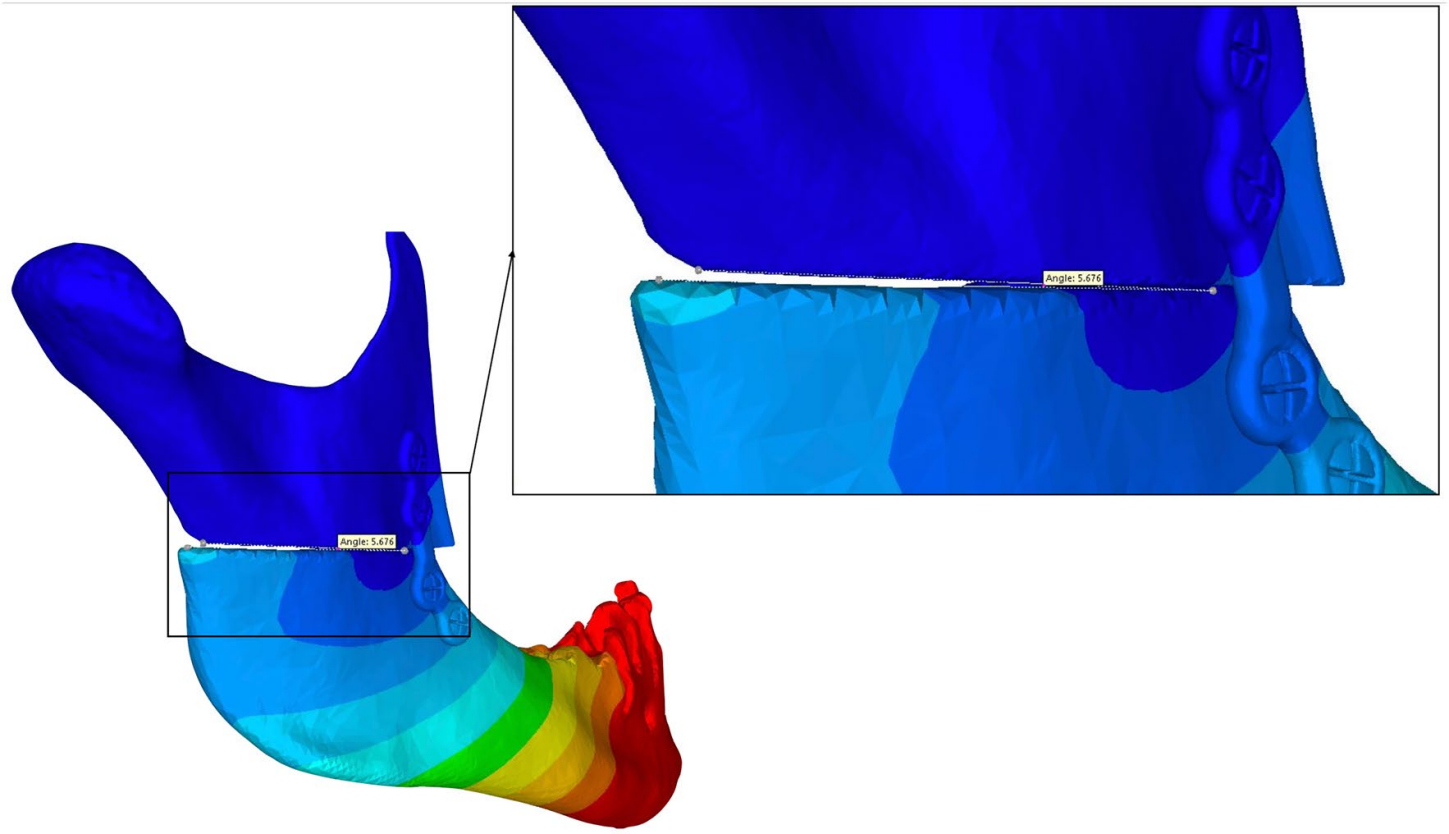
A gap with a 5° angle in the posterior area in the T2-A1 model shows a large displacement shift between the proximal and distal segment, compromising stability.

The smallest displacement in the T1 model was observed in the T1-A4 configuration, while the T2-A4 and T2-A5 configurations showed the lowest displacement in the T2 model. The highest displacements were observed in the T1-A1 and T2-A1 models. Moreover, a posterior gap of 5° was observed in the T2-A1 model, indicating a major shift displacement (Fig. 9).

## Discussion

Craniofacial surgery with skeletal fixation using plates and screws is used clinically to ensure stability despite gaps between bone segments. Securing stable fixation is a major concern in orthognathic surgery, in order to avoid non-union of bony fragments that compromise the outcome of surgery and lead to relapse. The type and location of fixations in HSOO are not well-defined, remain controversial, and depend on the approach exposure, handling of the short proximal segment, and clinical experience. It is critically important to optimize the fixations since HSOO reduces the contact area with bone compared to BSSO for equivalent displacement^9^, and therefore influences healing. Moreover the fixation method in BSSO^22^ and HSOO^7,10^ affects the temporomandibular joint position and may result in adverse effects. We have summarised in Table 2 the fixation configurations according to the main HSOO studies.

**Table 2:** Main HSOO clinical studies.

Authors	Number of patients (N) that underwent HSOO	Assessment	Type of fixation	Area of fixation	Intraoperative aid device(s)	Osteosynthesis complication(s)
Kaduch et al.^3^	17	Neurosensory alterations Amount of surgical displacement Bone healing	Standard mini-plates: double-Y and single-straight	One-third anterior and middle	Endoscope Condylar segment positioning plate	NR
Seeberger et al.^11^	50	Neurosensory alterations Function of the TMJ	Dedicated double mini-plate	One-third anterior and middle	None	NR
Seeberger et al.^8^	22	Condylar positioning	Dedicated double mini-plate	One-third anterior and middle	condylar positioning with mobile cone-beam tomography	NR
Landes et al.^11^	56	Skeletal stability Neurosensory alterations Amount of surgical displacement	Standard (X, Y straight) mini-plates × 2 (n = 23) Dedicated double plate (n = 33)	One-third anterior and middle	Condyle monitoring and positioning with sonography	2 standard mini-plate fractures
Kuehle et al.^11^	50	Condylar positioning	Dedicated double mini-plate	One-third anterior and middle	None	NR
Berger et al.^10^	10	Condylar positioning	Dedicated double mini-plate	One-third anterior and middle	Electromagnetic navigated condylar positioning	NR

NR, not reported; TMJ, temporo-mandibular joint.

**Figure 10:**
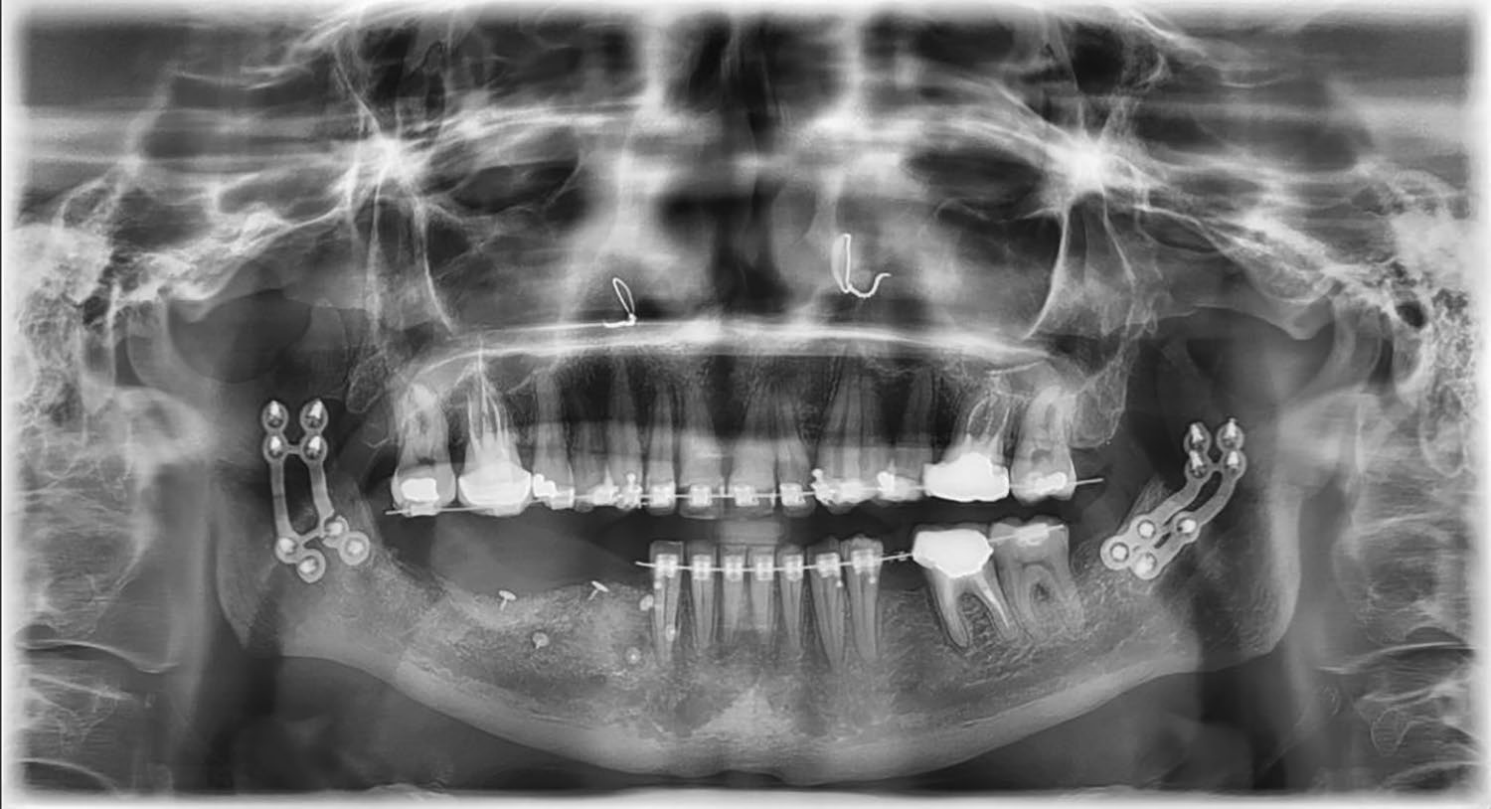
A fixation fracture that occurred in our clinical experience with a double-plate in the anterior position.

All the authors described the use of osteosynthesis in the one-third anterior and middle positions, corresponding to the A4 configurations in our study. HSOO approach exposure remains challenging and osteosynthesis can be complicated due to the existence of an area that is too difficult to navigate surgically, and therefore some authors use an endoscope for this^3^. In this approach, the anterior and middle one-third positions remain the most easily accessible areas for performing osteosynthesis. Only one clinical study^5^ focused on plating configurations, since mini-plate fractures had occurred in the author’s own experience. The authors decided to develop a dedicated “orthognathics” osteosynthesis, but no biomechanical data are available on the influence of the configuration of the fixation pattern. However, the biomechanical assessment of the bending yield strength of the construct that affects fatigue failure is necessary to understand why and how the fracture of the fixation occurs, as mentioned previously by many authors^5^ and ourselves (Fig. 10). Using FEM, we were able to determine the mechanical impact of various designs of internal fixation in HSOO surgery^23^. In addition, one should aim to reduce the volume and quantity of any implanted material^24^. A design process focused on shape and design variables optimization can produce bone plates that meet these two criteria: maximum stability with a minimum amount of implanted material.

FORGE NxT 3.0 is not specifically oriented towards biological applications but the easy addition and sharing of new features such as dedicated constitutive models is sufficient to encompass the broad framework needed for biomechanics. FORGE NxT 3.0 uses linear and nonlinear implicit finite element frameworks designed specifically for analysis in computational solid biomechanics. HSOO is a case of computational solid biomechanics and the mathematical model is based on the governing equations of continuum mechanics associated with the boundary and initial conditions. FORGE NxT 3.0 provides a numerical method of analysis based on the mathematical models which requires numerical discretization, solutions algorithms, and convergence criteria as requested in computational biomechanics^25,26^. Several finite element studies have been carried out using Forge software and have been validated in the literature^18,20,27^.

Models were developed from patients previously cared with HSOO technique and achieving a class I level of dental occlusion with improved facial balance and proportions. Class II and class III deformities are commonly treated conditions in orthognathic surgery^28^ and the amount of displacement of each model was decided according to the original treatment planning that corresponds to the mean possible displacement in HSOO surgery due to the recommended area of contact^9^. The HSOO technique was performed using the off-site planning software SurgiCase (Materialise, Leuven, Belgium), an on-site additive manufacturing tool^29^ (cutting and drilling guide) and patient-specific implants (PSI) were suited perfectly to the new anatomy of the displaced mandible. Titanium alloys are widely used in orthognathic surgery due to the excellent combination of properties such as a moderately high specific strength and high fatigue life. Unlike PSI, standard fixations require adjusted bending of the plates during the operation, and may fail due to the generated residual stresses, which affect the mean stress in fatigue loading.

In our double-plates models, the design involves a double bridge as used in clinical practice which facilitate positioning during the operative period. The use of two plates joined by two bridges produced mechanical change than fixing two separate plates. Theoretically, the plates with bridges exhibit less displacement than unbridged plates and could be associated with modified stress values, but additional sensitivity tests must be performed to confirm this.

Several simplifications and assumptions were made regarding the material properties of the models. All structures in this study were considered as homogeneous, linear elastic, and isotropic. Even though in vivo bone is not actually isotropic^30–32^, the structure of cortical and cancellous bones is generally considered to be transversely isotropic^33–35^. This simplification can have some biomechanical consequences to the mandible under loading^36^.

Vertical loading as the boundary condition was assumed in our study as per most previous numerical finite element analysis^37–39^ and in vitro biomechanical assessments^12^ in BSSO studies. However, Ayali et al.^40^ showed the relevance of testing the mandible not only under anterior vertical loading conditions but also under posterior vertical, horizontal, and oblique loading conditions. The constraint applied to the condyle and the vertical force applied under the chin area used in this study extrapolated the clinical environment of a clenching jaw during the immediate post-operative period when patients are not allowed to chew. From a biomechanical perspective, we assumed condylar restraint to provide an assessment of stress distribution in clenching conditions (worst post-operative case scenario). However, the temporomandibular joint dissipates tension during mandibular closing/opening and results could be overestimated according to the latter conditions. The response of the temporomandibular joint to the applied stress reaches an equilibrium in order to concentrate the lower tensions around the plate, the screws and the bone. On the other hand, a rigid system with a constraining condyle naturally reduces movements between bone segments to reduce the risk of plate failure but concentrates higher tensions in the bone/screw junction.

The mechanism represented by the 100 N loading occurs in any patients with an unconscious oral habit of rhythmic, non-functional clenching and grinding of teeth while performing movements that are not part of the masticatory function, and that will result in abnormal stress in bones and fixations. Thus, this study simulated and analysed the worst clinical case to understand the maximal stress distribution in bones and fixations.

The results of this study provide suggestions for plate positioning. We examined six different mini-plate configuration schemes in this study. The T2-A1 model showed very high displacement and excessive stress on bone structures and fixations. This suggests that the design of these configurations makes their stiffness questionable, and that the expected deflection in the mini-plates would jeopardize the stability of the fixation. A correlation between stress and displacement values in the present study showed that high stress levels can lead to the formation of an excessive gap between osteotomised fragments, which can result in malunion or non-union. Whatever the displacement of the distal bone segment, the A5 fixation configuration, i.e., anterior one-third + posterior one-third configuration in both models, showed an optimal balance between bone and fixation stress distribution and displacement. Higher stresses were observed in other configurations, including with the double mini-plates in the anterior one-third and middle (A4), which are commonly used in clinical studies (Table 2). From a biomechanical point of view, the results of this study suggest a change of habit in the osteosynthesis of HSOO surgery by placing the fixation with a double mini-plate in one-third of the anterior and posterior areas (A5) of the ramus in order to guarantee bone stability. This solution seems to be optimal for patients with either Class II or Class III dento-skeletal malocclusion.

The aim of this study was to assess the optimal design of the fixation and the optimal positioning of the plate. Model 5A (double plate in anterior and posterior position) showed better performance both in stability and stress distribution but it is important not to disregard the other models tested. Indeed, their maximum von Mises stress values never exceeded the yield strength of titanium alloy (880 MPa), which would imply a higher risk of failure. However, these models reached a sufficient level of tension to induce significant strain levels in the plates that compromise the biomechanical properties of the bone segment stability.

## Conclusions

The T1-A5 and T2-A5 models showed an optimal balance of stress distribution and displacement of bone segments. According to the results of this study, the double mini-plate positioned at one-third of the anterior and posterior areas is recommended in HSOO surgery to provide stable fixation for both advancement or set-back displacements.

## Data Availability

The datasets generated during and/or analysed during the current study are available from the corresponding author.
